# Effect of *Trichoderma viride* on rhizosphere microbial communities and biocontrol of soybean root rot

**DOI:** 10.3389/fmicb.2023.1204688

**Published:** 2023-06-02

**Authors:** Peixin Gao, Kai Qi, Yujuan Han, Liguo Ma, Bo Zhang, Yueli Zhang, Xiumin Guan, Junshan Qi

**Affiliations:** ^1^Shandong Key Laboratory of Plant Virology, Institution of Plant Protection, Shandong Academy of Agricultural Sciences, Jinan, China; ^2^Institute of Vegetables, Shandong Academy of Agricultural Sciences, Jinan, China; ^3^Shandong Agricultural Technology Extension Center, Jinan, China

**Keywords:** soybean root rot, seed dressing, *Trichoderma viride*, chemical fungicide, rhizosphere microbial community, co-occurrence network

## Abstract

Biological seed dressing is a cost-effective means to protect plant roots from pathogens. *Trichoderma* is generally considered as one of the most common biological seed dressings. However, there is still a dearth of information on the effects of *Trichoderma* on microbial community of rhizosphere soil. High-throughput sequencing was used to analyze the effects of *Trichoderma viride* and a chemical fungicide on microbial community of soybean rhizosphere soil. The results showed that both *T. viride* and chemical fungicide could significantly reduce the disease index of soybean (15.11% for Trichoderma and 17.33% for Chemical), while no significant difference was observed between them. Both *T. viride* and chemical fungicide could affect the structure of rhizosphere microbial community, they increased the β-diversity of microbial community and significantly reduce the relative abundance of Saprotroph-Symbiotroph. Chemical fungicide could reduce the complexity and stability of co-occurrence network. However, *T. viride* is beneficial for maintaining network stability and increasing network complexity. There were 31 bacterial genera and 21 fungal genera significantly correlated with the disease index. Furthermore, several potential plant pathogenic microorganisms were also positively correlated with disease index, such as *Fusarium*, *Aspergillus*, *Conocybe*, *Naganishia*, and *Monocillium*. From this work, *T. viride* may be used as a substitute for chemical fungicide to control soybean root rot and be more friendly to soil microecology.

## 1. Introduction

Soybean is an important legume crop, but is susceptible to many diseases, such as root rot, charcoal rot, sclerotinia stem rot and soybean cyst. The majority of losses are attributed to microbial diseases. Root rot has become one of the most destructive soil-borne diseases of soybean, which causes yield reduction and serious economic losses. The soybean yield loss caused by root rot is 10–60%, or even no yield in serious cases (Hartman et al., 2015). Root rot has been listed as one of the destructive diseases impairing soybean yield because of its wide distribution, strong destruction and difficult control (Chang et al., 2018).

Seed dressing can control pathogens early and raise healthy plant stand, which is considered as a simple, economical and effective method to control root-rot disease. Many chemical fungicides are now formulated as seed dressings, the chemicals are delivered into the soil along with the seed, which can reduce the number of pathogens or inhibit their reproduction, and protect germinating seedlings (Labanya et al., 2018). Although the use of chemical fungicides in plant disease management is acknowledged to be effective, many studies also have shown that chemical control of soil-borne pathogens is challenging because chemistries can adversely affect beneficial soil microbes to disrupt the ecological balance of rhizosphere microbial communities (Roth et al., 2020), and also pollutes the environment to threaten human health (Ali et al., 2021). Biological control of plant pathogens is a more promising method to control soil-borne diseases and is recognized by many people. *Trichoderma* is genetically very diverse, and it is also tolerant of a range of recalcitrant environmental pollutants (Tripathi et al., 2013). *Trichoderma* is well documented as potential growth promoting and effective biological control agents for many crop plants (Verma et al., 2007; Savazzini et al., 2009; Ferreira and Musumeci, 2021). *Trichoderma* populations can be established relatively easily in several types of soil and can continue to existence for months, because they grow rapidly and also because of their abundant conidiation (John et al., 2010; Tripathi et al., 2013). Biological control agents control soil-borne diseases mainly through competition with pathogens, parasitism and producing antifungal compounds (Verma et al., 2007; Savazzini et al., 2009). Owing to its antagonistic effect on inimical organism, *Trichoderma viride* ranks as one of the most potential biocontrol agents against several soil and seed borne pathogens (Bayoumi et al., 2019; Khan et al., 2021).

Rhizosphere soil microbiomes can form complex interactions with plants and play an important role in the health of plants in natural environments, including nutrient uptake, growth promotion, stress tolerance and resistance to pathogens (Trivedi et al., 2020). A balanced and stable relationship between plants and rhizosphere microbial communities may be the key to maintaining plant health and inhibiting plant diseases. Understanding the community composition of rhizosphere microbial community and its response to chemical or biological control treatment is critical for root rot control. This work aims to explore the control effect of *T. viride* on soybean disease and the potential impact of *T. viride* on rhizosphere microbial communities, and provide theoretical basis for the application of *T. viride* in the sustainable management of plant diseases.

## 2. Materials and methods

### 2.1. Seed dressing treatment

The chemical fungicide Cruiser® seed dressing consisting of 22.6% thiamethoxam, 2.2% fludioxonil and 2.2% difenoconazole (Syngenta Group China, Beijing, China) applied at the ratio of 3.3 mL/kg seeds, according to manufacturer’s instructions. *T. viride* spores were applied at the rate of 15 g/kg seeds and appropriate amount of sterile water was added to the group. The *T. viride* spores (1 × 10^8^ CFU/g) were provided by the Ecology Institute of Shandong Academy of Sciences. The blank group was treated with sterile water at the ratio of 3.3 mL/kg seeds. Before sowing, the seeds and seed dressing were uniformly mixed by stirring repeatedly.

### 2.2. Experimental design and soil sample collection

The experiment was carried out at the research station in Shentang village (Jining City, Shandong Province). The region belongs to a warm temperate monsoon climate with sufficient rainfall and sunshine in summer, which is suitable for the growth of soybean crops. In June 2020, three plots were selected for experiment at the research station. And the area of each plot was 5 hectares. The experiment was designed with three seed-dressing treatments. The Control group was a control, which used the same amount of sterile water instead of seed coating agent. Chemical group was the chemical fungicide Cruiser® seed dressing. Trichoderma group was a *T. viride* seed dressing.

In this study, all treatments were carried out under the same weather and field management conditions. After 30 days of sowing, 5 quadrats (5 × 5 m) were randomly selected in each plot, and 5 sampling points were selected by the diagonal method for each plot. Soybean plants and root-attached soil were collected at each sampling point for collection of rhizosphere soil samples and observation of soybean root rot disease. And 5 samples in each quadrat were taken as one parallel sample. The rhizosphere soil samples were collected according to the description of Bulgarelli et al. (2012). Briefly, shaken off the loosely attached soil, and then collected *ca.* 1-mm of soil covering the roots with sterile brush. After removing plant debris, the collected soil samples were stored at −80°C.

### 2.3. Evaluation of disease index and growth characteristic of soybean

The disease index, plant weight and plant height of soybean were evaluated by 50 plants randomly collected from each treatment. At harvest stage, soybeans were harvested from 3 plots (50 m^2^) from each experimental field to determine the yield. And the seed weight and pod number per plant of soybean were determined with 50 plants randomly collected from each treatment. The disease severity scale of soybean was evaluated according to the section “Fungicides control soybean root rot” in GB/T 17980.88–2004 pesticide field efficacy test guidelines (Grade 0 = no diseased spots on the stem and taproot of the plant; Grade 1 = a few disease spots on the stem and taproot; Grade 3 = many diseased spots on the stem or taproot, with the diseased spot area accounting for 1/4 to 1/2 of the total area of the stem and root; Grade 5 = multiple and large diseased spots on the stem and taproot, with the diseased spot area accounting for 1/2 to 3/4 of the total area of the stem and root; Grade 7 = disease spots on the stem or taproot are connected into patches, forming a phenomenon of wrapping around the stem, but the root is not dead; Grade 9 = root necrosis, plant aboveground parts withering or death), and then the disease index was calculated based on the above evaluation results. The disease index was calculated as follows:


$$\text{Disease index}(\%)=\frac{\sum \left(\text{disease rate}\times \text{number of plant with this rate}\right)}{\text{total number of plants}\times \text{maximum value of disease scale}}\times 100.$$

### 2.4. DNA extraction and sequencing

Total genomic DNA of rhizosphere soil was extracted using the FastDNA spin for soil kit (MP Biomedicals, USA) following the manufacturer’s instructions. All the DNA samples were stored at −20°C before sequencing. High-throughput sequencing was performed using an Illumina platform at the Novogene Biinformatics Technology Co., Ltd. For bacteria, the 16S rDNA was amplified using universal primers 515F/806R (5′-GTGCCAGCMGCCGCGGTAA-3′/5′-GGACTACHVGGGTWTCTAAT-3′). And for fungi, the second nuclear ribosomal internal transcribed spacer (ITS1) region was amplified using primers ITS1F/ITS2 (5′-CTTGGTCATTTAGAGGAAGTAA-3′/5′-GCTGCGTTCTTCATCGATGC-3′). Raw sequences have been stored in the NCBI Sequence Read Archive under the BioProject accession number PRJNA951875. Sequences were analyzed with the QIIME (Version 1.7.0) data analysis package (Caporaso et al., 2010). Generated operational taxonomy units (OTUs) were defined by clustering at a similarity threshold of 97%.

### 2.5. Statistical analysis

All microbial community 16S rDNA and ITS amplicon sequencing data analyses were carried out in R 4.1.3 based on normalized OTUs table. Bacterial and fungal alpha diversity indices (including the OTU richness, Shannon and phylogenetic diversity indices) were calculated using the “vegan” and “picante” packages in the R statistical computing environment. The statistical significance was analyzed using analysis of variance (ANOVA) followed by Tukey’s honest significant difference (HSD) *post hoc* tests, and *p <* 0.05 was regarded as significant. Nonmetric multidimensional scaling (NMDS) analysis was conducted using the metaMDS functions from the R package “vegan” with the Bray-Curtis metric. The Bray–Curtis distance was calculated using the vegdist function in the “vegan” package. Three nonparametric statistical tests were applied to determine the differences in microbial community structure of three treatments, including permutational multivariate analysis of variance (Adonis), analysis of similarity (ANOSIM), and multiple response permutation procedure (MRPP). And values were considered statistically different when *p <* 0.05. FUNGuild^1^ database was applied to predict fungal functions (Nguyen et al., 2016). According to the annotation results obtained by FUNGuild software, the confidence scores of ‘Highly Probable’ and ‘Probable’ were accepted in this study (Chen et al., 2020). The relative abundances of each trophic mode group for the three treatments were compared using a heat map. Similarity and clustering relationships of distinctive OTUs for each treatment were presented in a Venn diagram. Spearman’s correlation for abundant genera and disease indices were calculated using the corr. Test function in the R package “psych.” To quantify the assembly processes of microbial community, the nearest taxon index (NTI) was calculated by the null-model theory using the R package “picante” (Kembel et al., 2010), followed by the beta nearest taxon index (βNTI) for pairwise phylogenetic turnover between communities (Guan et al., 2021). βNTI values >2 or <−2 indicate the dominance of stochastic processes in shaping the community composition, whereas βNTI values between −2 and 2 indicate the dominance of deterministic processes (Dini-Andreote et al., 2015). To further identify the OTUs preferentially associated with seed-dressing treatments, the “DESeq2” package in R based on the negative binomial distribution was used to compute log 2-fold changes (log2FC) of OTUs. The OTUs were selected only when the adjusted *p* value was <0.05 and the absolute log2FC was >1 from the DESeq2 analysis. For all comparisons and statistical tests, the Benjamini–Hochberg approach was applied to adjust the *p*-values, and alpha = 0.05 was set as the significance threshold (Kunz et al., 2019).

### 2.6. Microbial network analysis

Co-occurrence network analysis was performed following Molecular Ecological Network Analyses Pipeline to elucidate the effects of different treatments on the overall microbial ecological network (Deng et al., 2012). The top 304 genera (>0.01% per samples) were retained and the genera with low frequencies (present in <3 samples) were removed for analysis to improve the reliability of network construction. After threshold scanning through RMT-based approach, the networks were constructed with an identical similarity threshold (S_t_ = 0.97) (Deng et al., 2012). Gephi was used to visualize the co-occurrence networks. The connectivity of each node was determined based on within-module connectivity (Zi) and among-module connectivity (Pi) (Guimera and Nunes Amaral, 2005). The potential keystone genera were defined based on the values of Zi and Pi, with thresholds of 2.5 and 0.62, respectively (Wu et al., 2022). The natural connectivity was used to evaluate network robustness to assess the difference in network stability for the three treatments (Pan et al., 2021). The network stability is derived by removing nodes in the static network to assess how quickly robustness degraded (Peng and Wu, 2016; Fan et al., 2018).

## 3. Results

### 3.1. Disease index and growth characteristic of soybean

**Figure 1:**
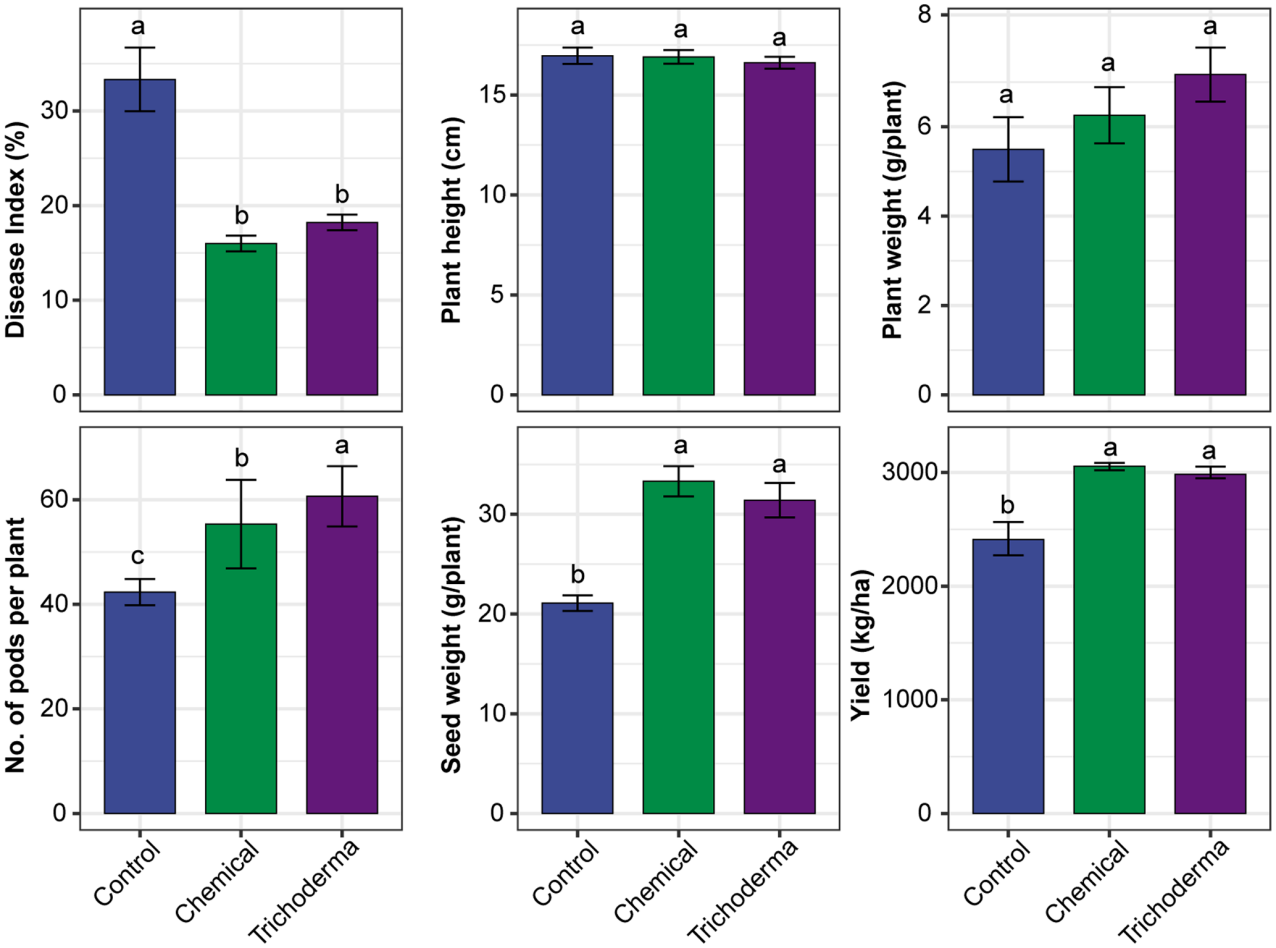
Effects of different treatments on the disease index and growth characteristic of soybean. Different letters indicate significant difference between treatments (*p* < 0.05).

The three treatments (Control, Chemical and Trichoderma) had different effects on the disease index and growth characteristic of soybean (Figure 1). The disease index of Control was 28.89–44.44%, while that of Chemical and Trichoderma were 13.33–17.78% and 15.56–20.00%, respectively. The disease index of both Chemical and Trichoderma were significantly lower than that of the Control (*p* < 0.05). The disease index of Trichoderma was slightly higher than that of the Chemical, but no significant difference was observed between the two treatments. No significant differences in the height and weight of soybean plants were observed among the three treatments. At the harvest stage, the pod number and seed weight per plant increased significantly in Chemical and Trichoderma, and the pod number of Trichoderma was significantly higher than that of Chemical. The soybean yield increased by 26.77 and 23.79% in Chemical and Trichoderma, respectively.

### 3.2. Alpha diversity analysis of microbial communities

Statistical significance analysis of alpha diversity indicated that the bacterial diversity among the three treatments was not significantly different (Table 1). Analysis of fungal alpha diversity showed that all alpha diversity indices in Trichoderma had no significant difference with the control, while the phylogenetic diversity and OTUs richness indices in Chemical were significantly higher than those in Control and Trichoderma (*p* < 0.05). In summary, the effect of *T. viride* on the alpha diversity of rhizosphere microbial community was limited, while chemical fungicide changed the richness and phylogenetic diversity of rhizosphere fungal community.

**Table 1:** Comparison of alpha diversity indices of rhizosphere bacterial and fungal communities under different treatments (Control, Chemical, and Trichoderma).

Comparisons	Treatment	OTUs richness	Shannon	Phylogenetic diversity
Bacteria	Control	1333.60 ± 32.58a	6.41 ± 0.02a	73.80 ± 1.24a
	Chemical	1348.80 ± 57.62a	6.36 ± 0.08a	72.93 ± 2.45a
	Trichoderma	1263.20 ± 60.24a	6.27 ± 0.08a	70.25 ± 2.47a
Fungi	Control	192.60 ± 8.82b	3.82 ± 0.08a	36.26 ± 0.88b
	Chemical	224.00 ± 7.33a	4.01 ± 0.10a	42.06 ± 1.19a
	Trichoderma	176.00 ± 5.55b	3.91 ± 0.06a	34.52 ± 0.90b

Results show Mean ± SE. Different letters indicate significant difference between treatments (*p* < 0.05).

### 3.3. Beta diversity analysis of microbial communities

**Figure 2:**
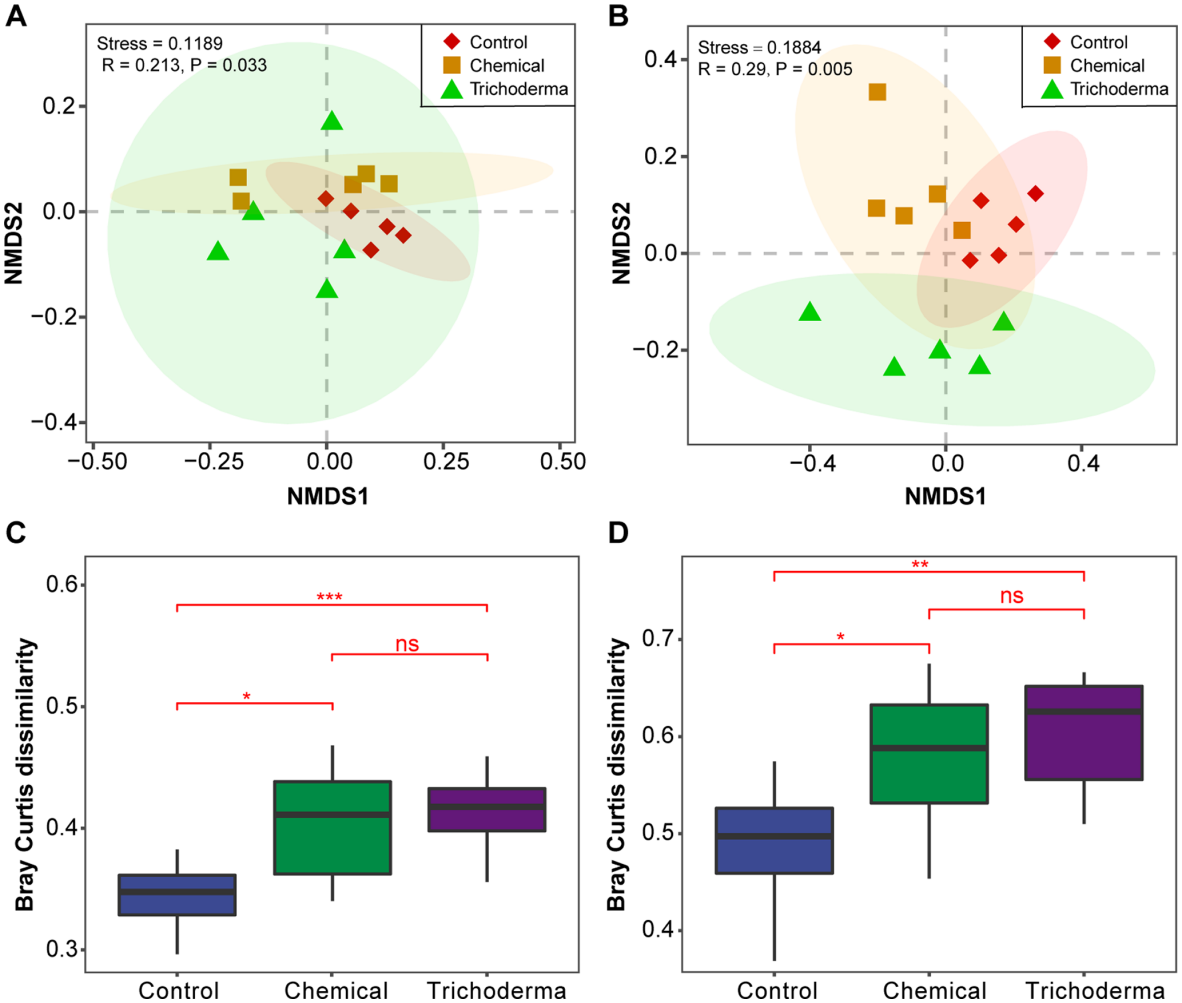
Beta diversity analysis of microbial communities. NMDS ordination of the Bray–Curtis dissimilarity of bacterial **(A)** and fungal **(B)** communities, Confidence ellipses level at 0.95. Effect of different treatments on bacterial **(C)** and fungal **(D)** communities dissimilarity, “ns” no significance, *p < 0.05, **p < 0.01, ***p < 0.001.

The bacterial and fungal community structure of the different treatments was visualized using the NMDS analysis. The soil samples of three treatments (Control, Chemical and Trichoderma) did not form significantly different clusters in the ordination space (Figures 2A,B). The analysis of the Bray–Curtis dissimilarity showed that there was no significant difference in the *β*-diversity of the microbial communities between the Chemical and Trichoderma treatments (Figures 2C,D). And the *β*-diversity of bacterial and fungal communities was significantly higher in the Chemical and Trichoderma treatments than that in the Control (*p* < 0.05). The non-parametric multivariate statistical tests, Adonis, ANOSIM and MRPP at the OTUs level indicated significant differences in microbial community among the three treatments (*p <* 0.05 in all cases) (Supplementary Table S1), and the differences among fungal communities were more significant (*p <* 0.01).

Stochastic and deterministic were two types of ecological processes that influence the microbial community assembly. To compare the relative importance of the two processes of the rhizosphere microbial community of each group, we calculated the βNTI values of bacterial and fungal communities, respectively (Supplementary Figure S1). Both processes drove the assembly of microbial community composition in soybean rhizosphere. Deterministic was the main force that influenced the bacterial community assembly (80% for Control, 70% for Chemical and 70% for Trichoderma) (Supplementary Figure S1A). However, the majority of the βNTI values for the rhizosphere fungal community compositions fell between −2 and 2 in the three treatments (Supplementary Figure S1B), suggesting that stochastic processes dominated the assembly of the rhizosphere fungal community compositions. In general, neither Chemical nor Trichoderma alter the assembly process of the microbial community.

### 3.4. Microbial community composition

**Figure 3:**
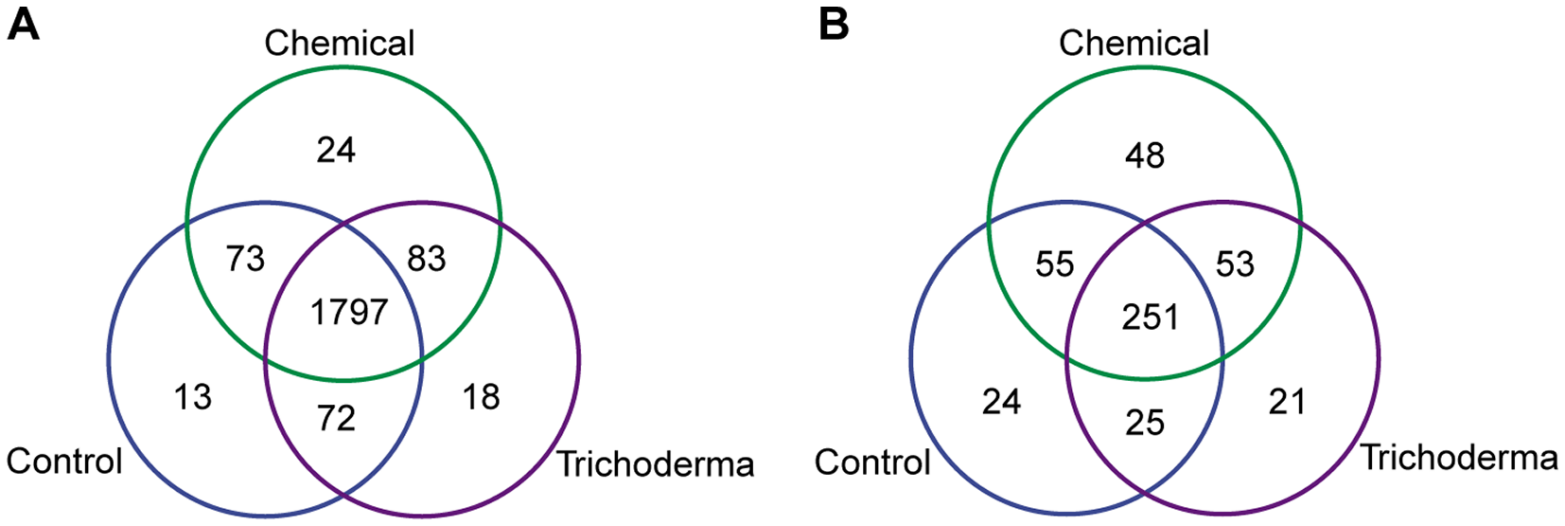
Venn diagram for the number of shared and unique bacterial **(A)** and fungal **(B)** OTUs under different treatments.

A total of 2080 bacterial OTUs and 477 fungal OTUs were identified from all samples. For bacteria, a total of 1950, 1977, and 1970 OTUs were found from the samples derived from Control, Chemical and Trichoderma, respectively. For fungi, 355, 407, and 350 OTUs were found from the three treatments, respectively (Figure 3). The majority of OTUs were shared by three treatments. And more unique OTUs were identified in Chemical treatment, indicating that the chemical fungicide has a stronger stimulating effect on bacterial and fungal communities.

**Figure 4:**
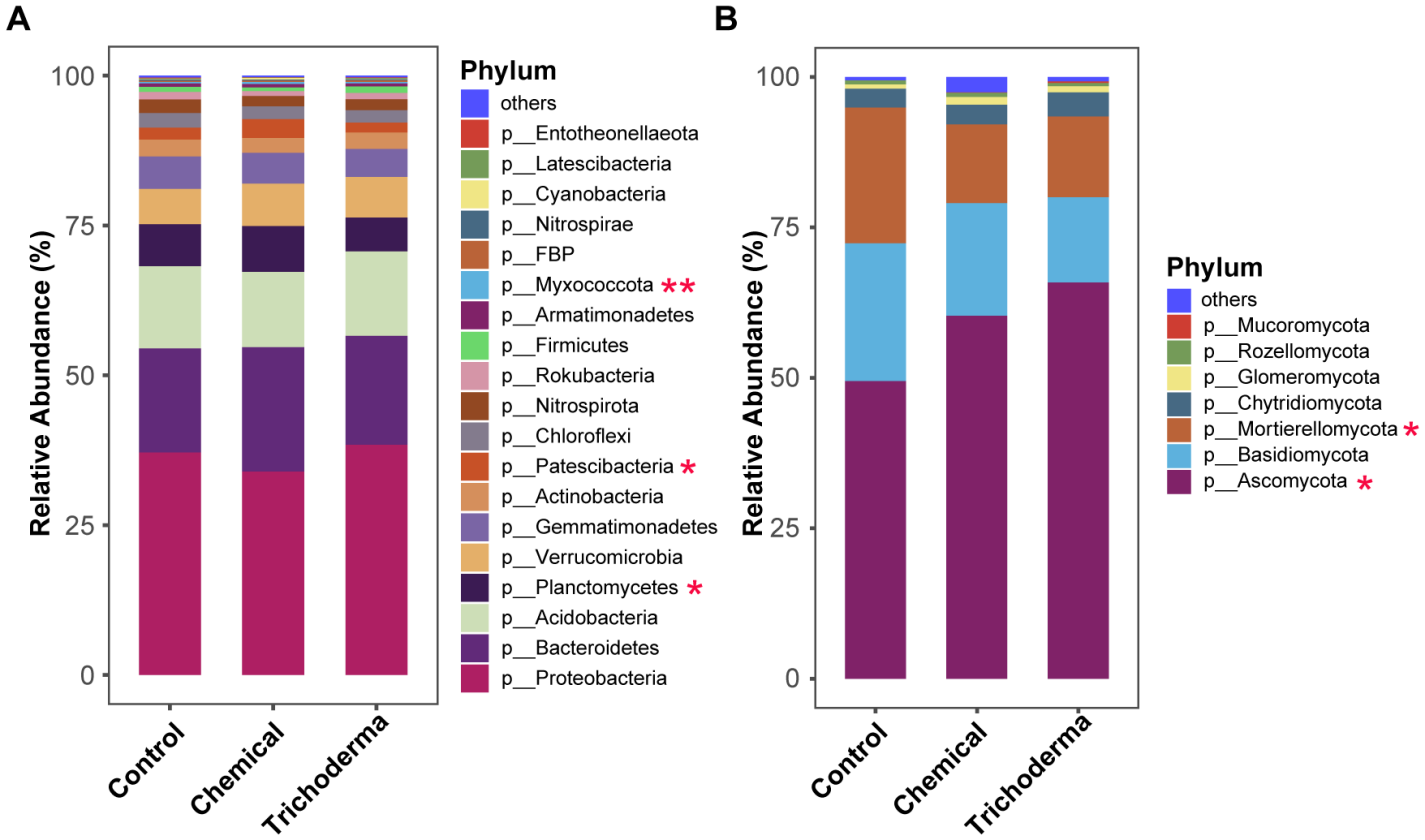
Relative abundance of bacterial **(A)** and fungal **(B)** communities at the phylum level of rhizosphere soil under different treatments (Control, Chemical and Trichoderma). “Others” represent the phyla with a relative abundance of less than 0.1%. *p < 0.05, **p < 0.01.

The analysis of the relative abundance of dominant bacteria and fungi (relative abundance >0.1%) at the phylum level indicated that both *T. viride* and chemical fungicide had influence on the microbial community composition (Figure 4). The dominant OTUs of bacterial in the three treatments belonged to 19 phyla, while those of fungi belonged to 7 phyla. *Proteobacteria* and *Ascomycota* were the most dominant phylum of bacteria and fungi, with relative abundances of 33.93–38.38% and 49.44–65.85%, respectively. For the bacterial community, statistical analysis showed that *Planctomycetes*, *Patescibacteria*, and *Myxococcota* were significant different among the three treatments (ANOVA, *p <* 0.05). For the fungal community, *Ascomycota* and *Mortierellomycota* were significant different among the different treatments (ANOVA, *p <* 0.05). The relative abundance of *Ascomycota* and *Myxococcus* in the Control were lower than those of the other two treatments, while *Mortierellomycota* was higher. And the *Planctomycetes* and *Patescibacteria* were found maximum in Chemical and minimum in Trichoderma.

**Figure 5:**
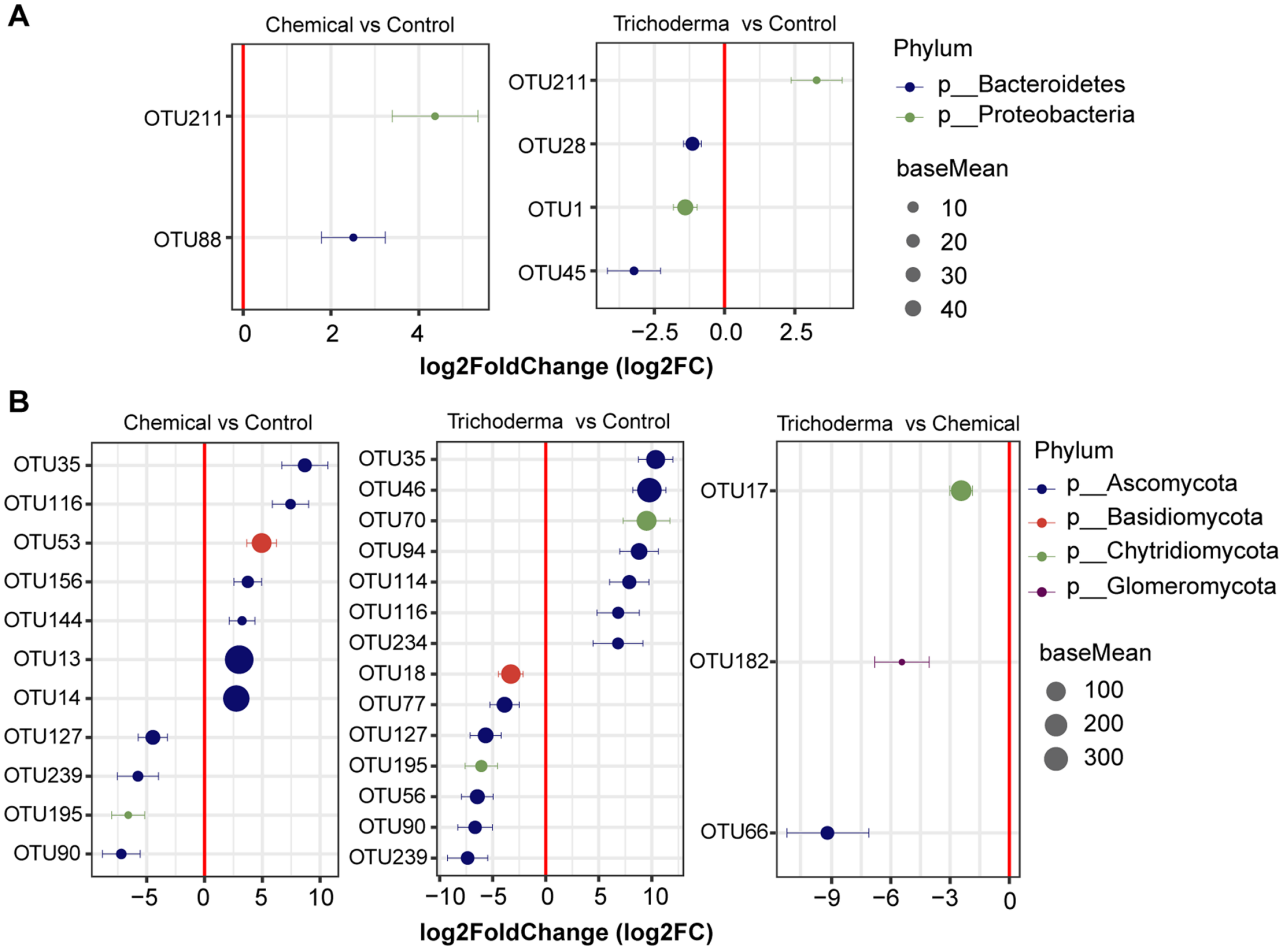
Differentially abundant bacterial **(A)** and fungal **(B)** OTUs identified in the rhizosphere soil from different treatments. The absolute log2FC was >1 and FDR adjusted *p*-values of <0.05 were considered to be differentially abundant.

From the result of DESeq2 algorithm, the fungal community was more sensitive to chemical fungicide and *T. viride* than bacterial community (Figure 5). There were 12 OTUs and 18 OTUs were influenced by chemical fungicide and *T. viride* treatments, respectively. And among them, 9 and 6 OTUs were significantly correlated with the disease index of soybean, respectively. In the Chemical, the relative abundance of 2 bacterial OTUs and 7 fungal OTUs increased, while that of 4 fungal OTUs decreased. In the Trichoderma, 1 bacterial OTUs and 7 fungal OTUs were enriched, while 3 bacterial OTUs and 7 fungal OTUs were reduced. These OTUs with significant differences in relative abundance belonged to *Proteobacteria*, *Bacteroidetes*, *Ascomycota*, *Basidiomycota*, or *Chytridiomycota* (Figure 5). Especially, 1 bacterial OTUs and 2 fungal OTUs were enriched in both Chemical and Trichoderma, and 4 fungal OTUs were significantly reduced (absolute log2FC > 1, *padj* < 0.05), which belonged to *Proteobacteria*, *Ascomycota and Chytridiomycota*. Most of the identified OTUs belonged to *Ascomycota*, indicating that *Ascomycota* may be more associated with soybean root rot and is easily affected by control measures. In addition, only 3 fungal OTUs were significantly enriched in Chemical compared to Trichoderma, and there was no significant difference in the relative abundance of bacterial OTUs between Chemical and Trichoderma.

### 3.5. FUNGuild analysis of fungal communities

**Figure 6:**
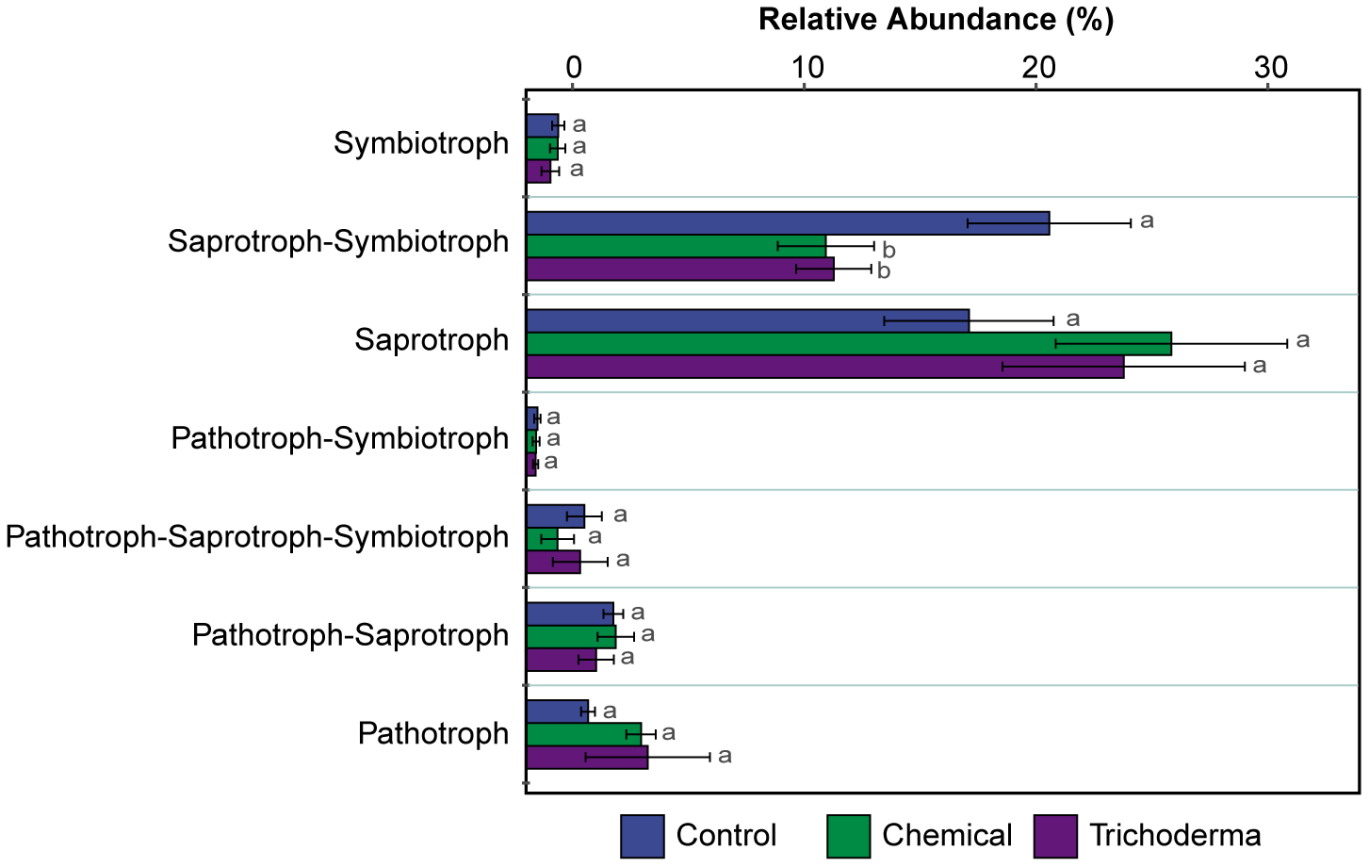
The relative abundance of trophic modes assigned by FUNGuild for fungal communities. Different letters indicate significant difference between treatments (*p <* 0.05).

FUNGuild was used to predict the nutritional and functional groups of the fungal communities. The results of FUNGuild showed that seven trophic modes could be classified, including Pathotroph, Pathotroph-Saprotroph, Pathotroph-Saprotroph-Symbiotroph, Pathotroph-Symbiotroph, Saprotroph, Saprotroph-Symbiotroph and Symbiotroph (Figure 6). Of the total 286 (59.96%) fungal OTUs assigned to trophic modes, only 224 (46.96%) OTUs had an assignment with highly probable and probable confidence ranks. The Saprotroph and Saprotroph-Symbiotroph were the major fungal guilds, with the OTUs percentages being 19.09–27.82% and 12.92–22.55%, respectively (Figure 6). The relative abundance of Saprotroph-Symbiotroph in Chemical and Trichoderma treatments (12.92 and 13.26%, respectively) was reduced compared to Control (22.55%), while Saprotroph showed the reverse trend. Statistical analysis showed that only the difference of Saprotroph-Symbiotroph among the three treatments was significant (*p* < 0.05).

### 3.6. Co-occurrence network analysis of microbial communities

**Figure 7:**
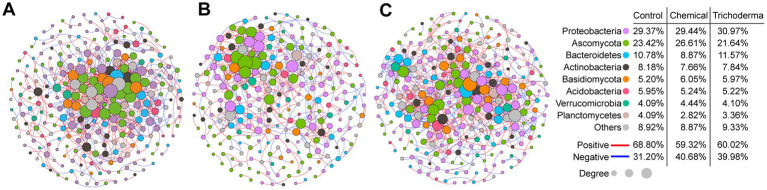
Co-occurrence networks of microbial genera in Control **(A)**, Chemical **(B)**, and Trichoderma **(C)**. The size of each node is proportional to the number of connections (degree). The color of connections between two nodes represents a positive (red) or a negative correlation (blue). The node colors show various phyla.

The microbial co-occurrence networks of Control, Chemical and Trichoderma consisted of 269, 248, and 268 nodes (genera), respectively (Figure 7). Most nodes of the three networks were primarily assigned to *Proteobacteria* (29.37% for Control, 29.44% for Chemical and 30.97% for Trichoderma). *Ascomycota* (23.42% for Control, 26.61% for Chemical and 21.64% for Trichoderma) and *Basidiomycota* (5.20% for Control, 6.05% for Chemical and 5.97% for Trichoderma) were the most abundant fungal nodes. Specifically, the proportion of fungal nodes in the network of the Chemical (35.89%) was higher than that of the Control (30.86%) and Trichoderma (30.22%). A total of 766, 499 and 818 co-occurrence relationships were inferred for the consensus microbial network of Control, Chemical and Trichoderma, respectively. Compared with the Control (68.80%), both Chemical (59.32%) and Trichoderma (60.02%) had less positive interaction in the microbial community, suggesting that they exhibited stronger competition among genera. Network degree for microbial nodes obeyed a power-law distribution (*p* < 0.001 in all cases) (Supplementary Figure S2), indicating non-random community assembly may be the characteristic of the three microbial communities (Barberan et al., 2012).

Multiple network topological metrics revealed that the microbial networks of the three treatments have different complexity and stability (Table 2). Compared with the Control network, Trichoderma had higher parameters of edge numbers, modularity, graph density, average degree and average clustering coefficient, and a lower parameter of average path distance. However, the average path distance of Chemical was higher than that of the control, while the node numbers, edge numbers, graph density and average degree were lower than that of the control. The high modularity (0.616 for Control, 0.736 for Chemical and 0.648 for Trichoderma) of the three networks illustrated that they all have modular structures (modularity values >0.4) (Newman, 2006; Ju and Zhang, 2015).

**Table 2:** Topological properties of networks associated with soybean rhizosphere microbiome under different treatments.

Network metrics	Control	Chemical	Trichoderma
Nodes	269	248	268
Edges	766	499	818
Modularity	0.616	0.736	0.648
Graph density	0.021	0.016	0.023
Average degree	5.695	4.024	6.104
Average clustering coefficient	0.234	0.327	0.314
Average path distance	4.733	5.068	4.67

Most genera identified in the three treatments were peripherals (97.40% for Control, 94.35% for Chemical and 95.90% for Trichoderma), which have most of their links inside their modules (Supplementary Figure S3A). Only 2.6, 5.65 and 4.1% of the genera in Control, Chemical and Trichoderma were designated as keystone genera, respectively (Pi >0.62 or Zi > 2.5). For Control, there were 3 module hubs, which are most closely related to *Actinobacteria* (2 genera) and *Proteobacteria* (1 genus), and 4 connectors belonging to *Actinobacteria* (2 genera), *Ascomycota* (1 genus) and *Proteobacteria* (1 genus). For Chemical, there were 9 module hubs belonging to *Ascomycota* (3 genera), *Proteobacteria* (3 genera), *Basidiomycota* (1 genus), *Bacteroidetes* (1 genus) and *Actinobacteria* (1 genus), and 5 connectors belonging to *Ascomycota* (4 genera) and *Proteobacteria* (1 genus). For Trichoderma, there were 8 module hubs belonging to *Ascomycota* (2 genera), *Basidiomycota* (3 genera), *Bacteroidetes* (1 genus), *Actinobacteria* (1 genus) and *Proteobacteria* (1 genus), and 3 connectors belonging to *Ascomycota* (1 genus), *Basidiomycota* (1 genus) and *Myxococcota* (1 genus). The relative abundance of keystone genera in Chemical and Trichoderma increased, most of which belonged to fungi. Moreover, the relative abundance of the keystone genera in Trichoderma was the highest (Supplementary Figure S3B).

**Figure 8:**
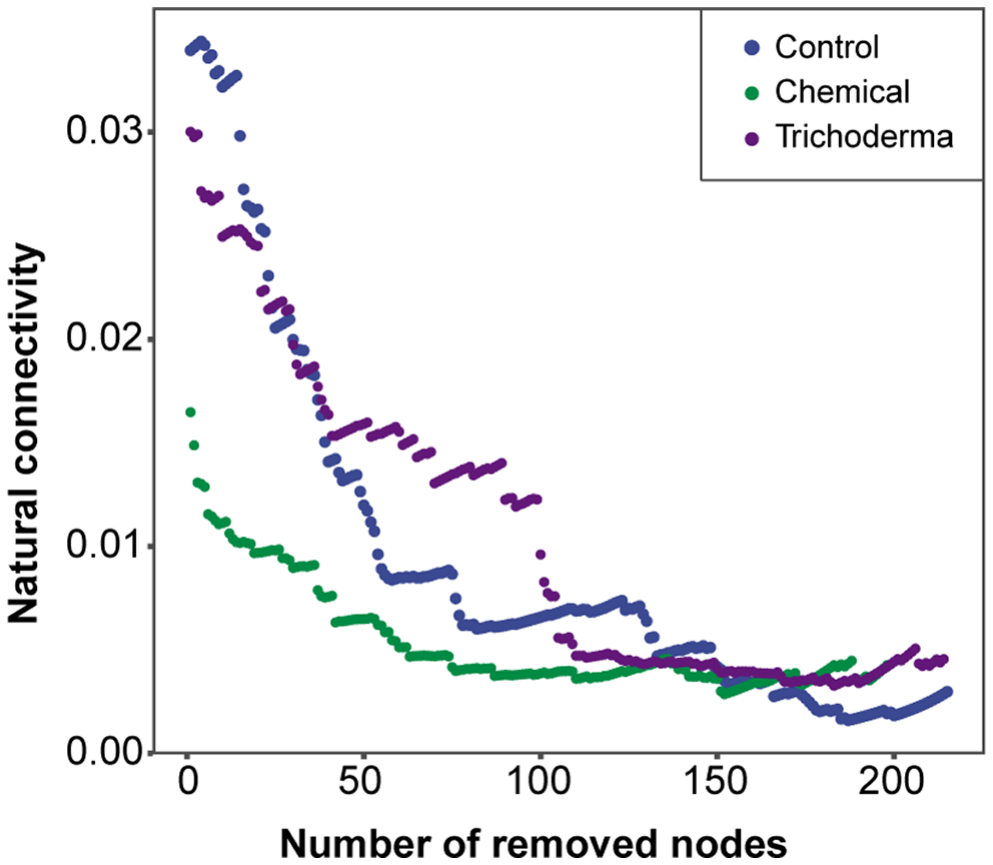
The natural connectivity of co-occurrence networks in three treatments.

The natural connectivity in all treatments gradually decreased with the increasing number of removed nodes (Figure 8). Compared with the Control network, the natural connectivity in the Chemical network was reduced, while that in the Trichoderma network was reduced little. The evidence implied that chemical fungicide may reduce the stability of microbial network, while *T. viride* does not. The higher network stability in Control may be due to the fact that these keystone genera (module hubs and connectors) influence the community structure through strong biotic interactions with the host or with other microbial taxa, rather than through their own high abundance (Supplementary Figure S3B and Figure 8).

### 3.7. Correlation between disease index and microbial communities

Associations between the abundant genera (abundance >0.1%) and disease index were analyzed by Spearman’s correlation coefficient (Supplementary Table S2). The relative abundance of 31 bacterial genera and 21 fungal genera were significantly correlated with the disease index of soybean, and there were 12 (38.71%) bacterial genera and 10 (47.62%) fungal genera positively correlated (*r* > 0, *p* < 0.05) with the disease index. For bacteria, the genera *Chiayiivirga*, *Salmonella*, *Rhodobacter*, *Mucilaginibacter*, *Pseudoxanthomonas*, *Pedobacter*, *Opitutus*, *Stenotrophobacter*, *Rhizobacter*, *Acidovorax*, *Kaistia*, *Neorhizobium*, *Acinetobacter*, *Dyadobacter*, *Oligoflexus*, *Microterricola*, *Lacunisphaera*, *Pantoea*, and *Asticcacaulis* were negatively correlated with the disease index, while the genera MND1, *Bradyrhizobium*, *Tumebacillus*, Pir4_lineage, UTBCD1, *Marmoricola*, WX53, *Blastopirellula*, *Duganella*, *Paraburkholderia*, *Caballeronia* and OLB17 showed a significant positive correlation. For fungi, the genera *Coprinellus*, *Schizothecium*, *Waitea*, *Sarocladium*, *Filobasidium*, *Limonomyces*, *Zopfiella*, *Acremonium*, *Symmetrospora*, and *Torula* were significantly negatively correlated with the disease index, while the genera *Lecythophora*, *Exophiala*, *Monocillium*, *Chrysosporium*, *Trichoderma*, *Conocybe*, *Tausonia*, *Fusarium*, *Coniochaeta*, *Aspergillus*, and *Naganishia* showed a significant positive correlation. Among the different genera that significantly correlated with the disease index, *Coprinellus* (*r* = −0.786, *p* < 0.001) exhibited an extremely significant negative correlation, while *Lecythophora* (*r* = 0.772, *p* < 0.001) showed an extremely significant positive correlation.

## 4. Discussion

### 4.1. *Trichoderma viride* reduced soybean root rot

*Trichoderma* species are used widely as biocontrol agents because they have many benefits on plant growth and have been proven to play a key role in controlling the plant diseases for several times (Sharma et al., 2011; Shahid and Khan, 2018). Among the various species of the *Trichoderma*, *T. viride* is considered to be one of the effective biocontrol agents for controlling root diseases of plants such as groundnut, mung bean, onion and so on (Manjula et al., 2004; Shahid and Khan, 2018; Bayoumi et al., 2019). The differences in control effects between studies may be related to the differences in plant types, regions and microbial communities (Li et al., 2020). In this study, the experimental results showed that *T. viride* and chemical fungicides reduced the disease incidence by 15.11 and 17.33% against soybean root rot, respectively (Figure 1). The control efficiency of *T. viride* was almost as high as that of chemical fungicide, and similar result had also been reported by Sui et al. (2022). Moreover, like other biocontrol agents, *T. viride* may also have the added benefit of protecting soil environment, improving plant growth and yield of soybean (Butt et al., 2001; John et al., 2010; Tan et al., 2017; Shahid and Khan, 2018).

### 4.2. The effects of *Trichoderma viride* on rhizosphere microbial community structure

The structure of soil microbial community has a crucial impact on soil quality and function (Janvier et al., 2007). The rhizosphere microbial community is also considered to be critical to improve crop productivity, resist soil-borne diseases, maintain soil ecosystem function and long-term sustainability (Trivedi et al., 2020). In the prevention and control of soybean root rot, special attention should be paid to the impact of the use of biological control agents and chemical fungicides on the rhizosphere microbial community (Wille et al., 2021). The rich root exudates have been shown to affect microbial communities in the rhizosphere soil (Butler et al., 2003). Pathogen attack can result in higher production of plant exudates, which contain proteins and extracellular DNA with antimicrobial functions (Basu et al., 2006; Minh Tran et al., 2016), thus influencing rhizobiome composition (Benizri et al., 2007). On the one hand, *Trichoderma* and chemical fungicides can directly affect rhizosphere microbial community. On the other hand, they can also indirectly affect the microbial community by affecting the production of plant root exudates (Howell, 2006).

In this study, *T. viride* did not significantly affect the alpha diversity of the rhizosphere bacterial and fungal communities compared to chemical fungicide that caused weakly significant differences in fungal richness (Table 1). This was similar to the effect of *Trichoderma* reported by Labanya et al. (2018) on the microbial community. On the contrary, Fu et al. (2019) and Sui et al. (2022) reported that treating soil with *Trichoderma* would elicit changes in soil microbial diversity. These effects may be related to the dosage of *Trichoderma* seed dressings (Liu and Hsiang, 1994). The microbial beta-diversity increased significantly in both Chemical and Trichoderma (Figure 2), implying that the decrease of soybean disease may be related to the increase of microbial beta-diversity. The differences between fungal communities were more significant than those of bacteria (Supplementary Table S1), which may be because the fungal community was more sensitive to *T. viride* and chemical fungicide than the bacterial community.

Exploring the formation and maintenance mechanisms of species diversity is an important scientific issue in soil microbial research (Nemergut et al., 2013). It is generally accepted that the rhizosphere microbial community is mainly profoundly shaped by metabolites produced by plant root (Bulgarelli et al., 2013). Consistent with previous studies on rhizosphere microbial community (Mendes et al., 2014; Fan et al., 2017), deterministic was the main force that influenced the bacterial community assembly (Supplementary Figure S1). However, the stochastic process in this study drove fungal community assembly. From this study, the fungal community may have the greater influence on the disease index of soybean root rot than bacterial community (Figure 5). The bacteria and fungi that changed significantly in the experiment may have a close relationship with the disease of soybean root rot. In this study, several potential plant pathogenic microorganisms were also positively correlated with disease index, such as *Fusarium*, *Aspergillus*, *Conocybe*, *Naganishia*, and *Monocillium* (Girlanda et al., 2001; Tekaia and Latgé, 2005; Liu et al., 2018; Horváth et al., 2020; Li et al., 2020). FUNGuild predicted the nutrient and functional groups of fungal community, which showed that the relative abundance of Saprotroph-Symbiotroph reduced in Chemical and Trichoderma treatments (Figure 6). Therefore, the Saprotroph-Symbiotroph in this work may play the most important role in soybean root rot.

### 4.3. The effects of *Trichoderma viride* on the co-occurrence network of microbial community

Hassani et al. have demonstrated that plant growth and fitness may be altered in beneficial ways by enhanced interactions within rhizosphere bacterial communities (Hassani et al., 2018). In this work, network analysis revealed the microbial community network of Trichoderma consisted of the most nodes and edges, whereas the microbial community network of Chemical comprised of the least nodes and edges (Table 2). We observed that *T. viride* and chemical fungicide increased the proportion of negative interactions in rhizosphere microbial network. The increase of negative interaction is helpful to improve the tolerance of rhizosphere microbial communities to external pressures, and thus promote plant performance and fosters plant growth (Coyte et al., 2015; de Vries et al., 2018). The high stability and complexity of the rhizosphere microbial network may be beneficial to enhance the resistance to disturbance and the soil ecosystem’s multifunctionality (Ding et al., 2023). The differential complexity reflects the difference of protection or destruction of seed dressings on rhizosphere microbial community structure. Previous studies also have suggested that fungicides can significantly reduce the microbial network complexity (Han et al., 2021, 2022; Zhang et al., 2021). While *T. viride* can enhance the complexity of microbial network to resist the attack of pathogens, which plays a key role in protecting soil ecology and sustainable agricultural development. The keystone genera have important ecological functions and play important roles in network structure, which can be identified as targets for microbial modulation to maintain crop health and improve productivity (Olesen et al., 2007; Fan et al., 2018). We found that more fungal genera were identified as keystone genera in the network of Trichoderma and Chemical, which may effectively enhance the resistance of soybean to pathogens (Supplementary Figure S3). In particular, chemical fungicide significantly reduced the stability of the microbial network, which may be a potential risk for the sustainable development of agriculture, while *T. viride* did not (Figure 8). In conclusion, compared with chemical fungicide, *T. viride* can enhance the complexity of rhizosphere soil microbial network, maintain the stability of the network, and be more beneficial to protect soil microecology.

## 5. Conclusion

This study compared the effects of *T. viride* and chemical fungicide on the control of soybean root rot, the diversity and composition of rhizosphere microbial community. The results indicated that *T. viride* and chemical fungicide could significantly reduce the disease index of soybean root rot and increase soybean yield. Both *T. viride* and chemical fungicide could increase the β-diversity of microbial community and significantly reduce the relative abundance of Saprotroph-Symbiotroph. Specially, our study showed that *T. viride* is beneficial to increase the complexity of co-occurrence network and maintain the stability of network, which is different from chemical fungicide. In addition, there were 31 bacterial genera and 21 fungal genera significantly correlated with the disease index (*p* < 0.05). Furthermore, the *Coprinellus* genus showed an extremely significant negative correlation, while *Lecythophora* showed an extremely significant positive correlation. For the prevention and control of soybean root rot, we may pay more attention to these microorgnisms that are significantly related to the disease index in the future. In summary, *T. viride* may be used as a substitute for chemical fungicide to control soybean root rot and create a healthier soil microecology.

## Data availability statement

The datasets presented in this study can be found in online repositories. The names of the repository/repositories and accession number(s) can be found at: https://www.ncbi.nlm.nih.gov/, PRJNA951875.

## Author contributions

JQ conceived the idea and designed the experiment. PG, KQ, LM, BZ, and YZ performed the experiment. XG collected the data and samples. PG and YH performed the data analyses and wrote the manuscript. PG, KQ, YH, LM, BZ, YZ, XG, and JQ reviewed the manuscript. All authors contributed to the article and approved the submitted version.

## Funding

This study was supported by the National Key R&D Program of China (2018YFD0201005).

## Conflict of interest

The authors declare that the research was conducted in the absence of any commercial or financial relationships that could be construed as a potential conflict of interest.

## Publisher’s note

All claims expressed in this article are solely those of the authors and do not necessarily represent those of their affiliated organizations, or those of the publisher, the editors and the reviewers. Any product that may be evaluated in this article, or claim that may be made by its manufacturer, is not guaranteed or endorsed by the publisher.

## Footnotes

^1^ https://github.com/UMNFuN/FUNGuild
