# Supplementary material for: Effect of Trichoderma viride on rhizosphere microbial communities and biocontrol of soybean root rot
Source: Front Microbiol. 2023 Jun 2;14:1204688. doi: 10.3389/fmicb.2023.1204688 (PMC10272447; doi:10.3389/fmicb.2023.1204688)
Supplement: Supplementary file 1 [file Data_Sheet_1.docx]

Supplementary Material

**Effect of *Trichoderma viride* on rhizosphere microbial communities and biocontrol of soybean root rot**

Peixin Gao, Kai Qi, Yujuan Han, Liguo Ma, Bo Zhang, Yueli Zhang, Xiumin Guan, Junshan Qi*

*** Correspondence:** Junshan Qi: [qi999@163.com](mailto:qi999@163.com)

**List Supplementary Materials:**

**Supplementary Figure S1.** Distribution of βNTI based on the different treatments. Positive (or negative) βNTI values indicate greater (or less) than expected turnover in phylogenetic composition. The horizontal dotted black line (above 2 or below -2 are statistically significant) shows the 95 % confidence intervals around the expectation under neutral community assembly.

**Supplementary Figure S2.** The network degree distribution patterns of microbial network degree in the rhizosphere.

**Supplementary Figure S3.** Distribution and relative abundance of genera based on their network roles. Network roles of analysing module feature at genera level with the composition of connectors and module hubs (A), the composition of keystone genera in rhizosphere soil (B).

**Supplementary Table S1.** Dissimilarity analysis of microbial community composition associated with rhizosphere soils, (Adonis, ANOSIM and MRPP tests). Bold values indicate test results with *p* < 0.05.

**Supplementary Table S2.** Spearman’s correlation analysis between microbial genus relative abundance and disease index. “r” the correlation coefficient, “g” genus. * *p* < 0.05, ** *p* < 0.01, *** *p* < 0.001.

# Supplementary Figures and Tables

## Supplementary Figures


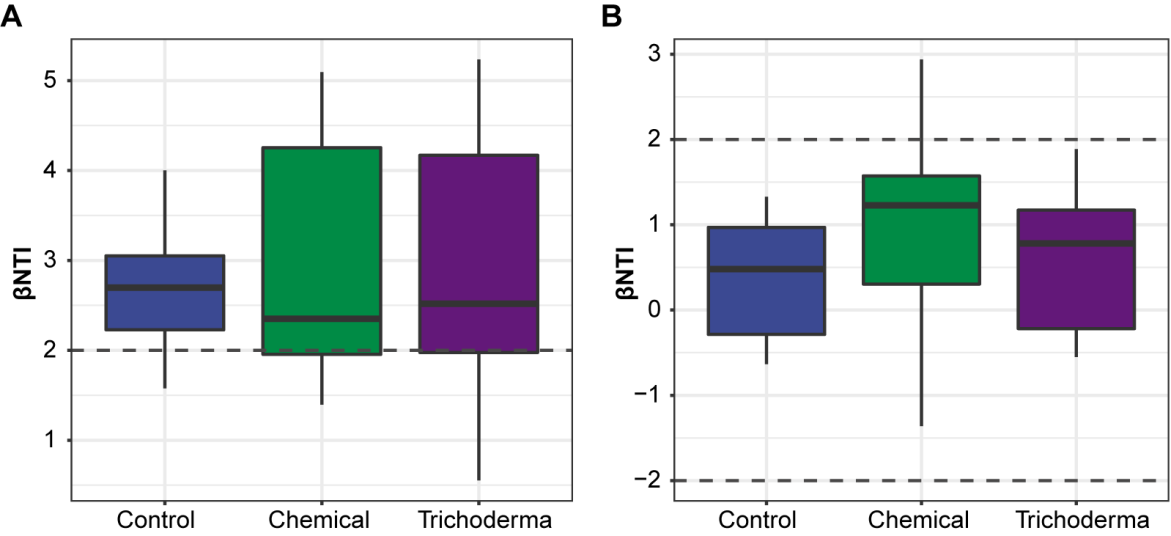


**Supplementary Figure S1.** Distribution of βNTI based on the different treatments. Positive (or negative) βNTI values indicate greater (or less) than expected turnover in phylogenetic composition. The horizontal dotted black line (above 2 or below -2 are statistically significant) shows the 95 % confidence intervals around the expectation under neutral community assembly.


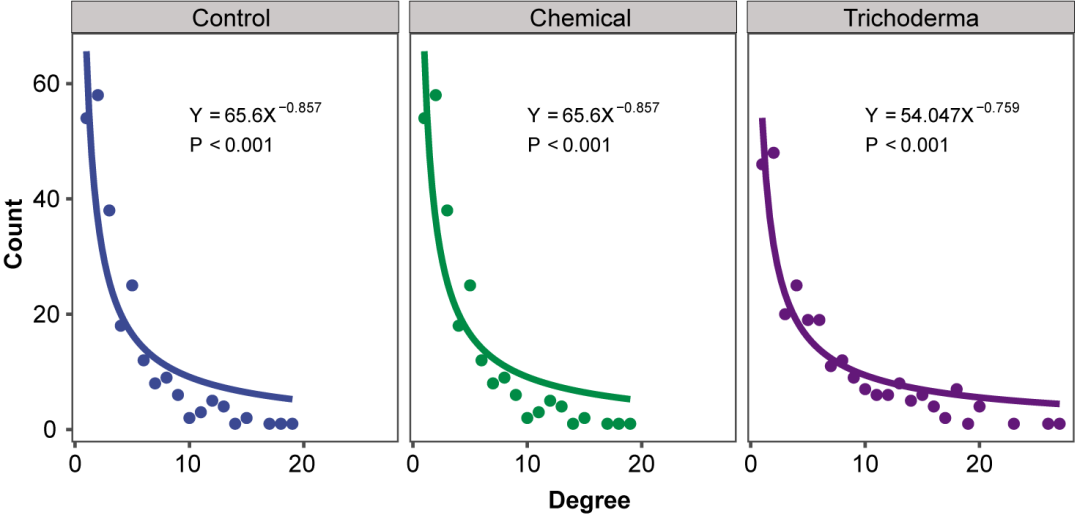


**Supplementary Figure S2.** The network degree distribution patterns of microbial network degree in the rhizosphere.


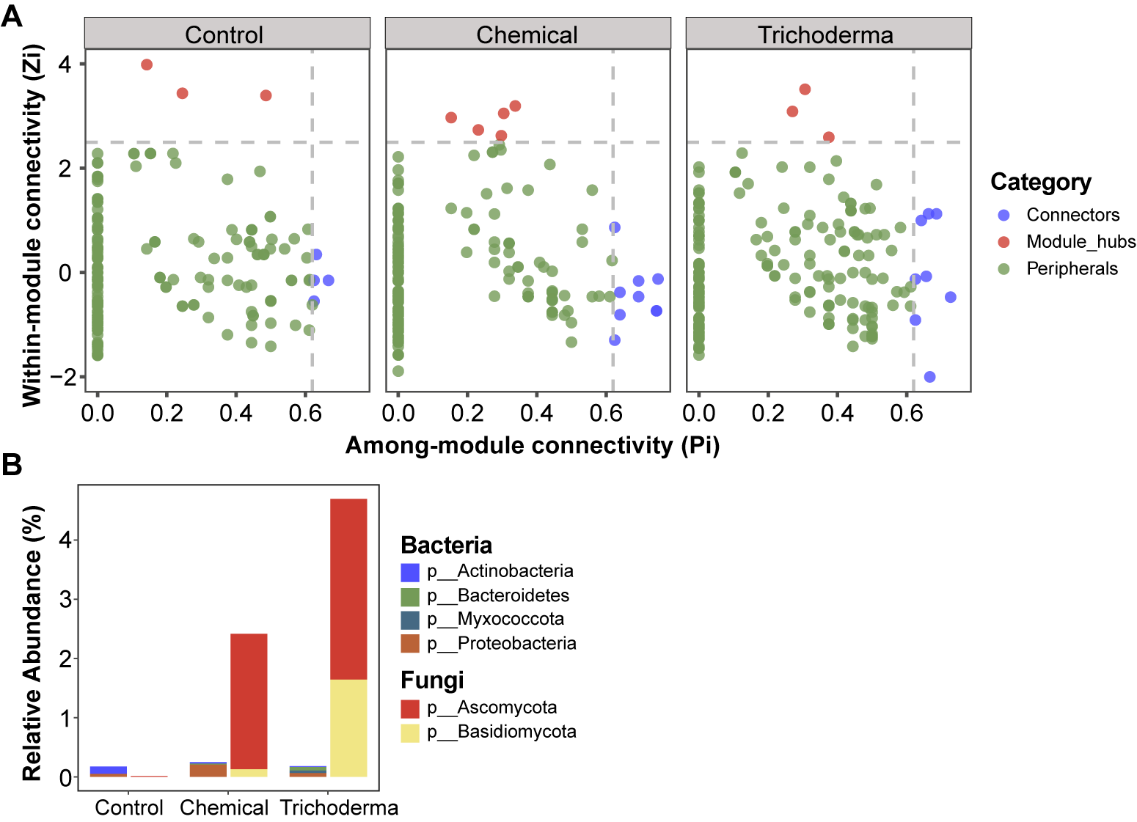


**Supplementary Figure S3.** Distribution and relative abundance of genera based on their network roles. Network roles of analysing module feature at genera level with the composition of connectors and module hubs (A), the composition of keystone genera in rhizosphere soil (B).

## Supplementary Tables

**Supplementary Table S1.** Dissimilarity analysis of microbial community composition associated with rhizosphere soils, (Adonis, ANOSIM and MRPP tests). Bold values indicate test results with *p* < 0.05.

| Comparisons |  | Adonis | | |  | ANOSIM | |  | MRPP | | |
| --- | --- | --- | --- | --- | --- | --- | --- | --- | --- | --- | --- |
|  |  | F | R^2^ | *P* |  | R | *P* |  | Observed δ | Expected δ | *P* |
| Bacteria |  | 1.488 | 0.199 | **0.036** |  | 0.213 | **0.033** |  | 0.388 | 0.401 | **0.04** |
| Fungi |  | 1.758 | 0.227 | **0.003** |  | 0.290 | **0.005** |  | 0.559 | 0.589 | **0.001** |

**Supplementary Table S2.** Spearman’s correlation analysis between microbial genus relative abundance and disease index. “r” the correlation coefficient, “g” genus. * *p* < 0.05, ** *p* < 0.01, *** *p* < 0.001.

| Genus  (Bacteria) | Disease Index | |  | Genus  (Fungi) | Disease Index | |
| --- | --- | --- | --- | --- | --- | --- |
|  | r | *p* |  |  | r | *p* |
| g__MND1 | 0.714** | 0.003 |  | g__*Coprinellus* | -0.786*** | 0.001 |
| g__*Chiayiivirga* | -0.665** | 0.007 |  | g__*Lecythophora* | 0.772*** | 0.001 |
| g__*Salmonella* | -0.655** | 0.008 |  | g__*Exophiala* | 0.72** | 0.002 |
| g__*Rhodobacter* | -0.654** | 0.008 |  | g__*Monocillium* | 0.705** | 0.003 |
| g__*Bradyrhizobium* | 0.645** | 0.009 |  | g__*Chrysosporium* | 0.676** | 0.006 |
| g__*Tumebacillus* | 0.632* | 0.012 |  | g__*Trichoderma* | 0.615* | 0.015 |
| g__*Mucilaginibacter* | -0.63* | 0.012 |  | g__*Conocybe* | 0.609* | 0.016 |
| g__Pir4_lineage | 0.622* | 0.013 |  | g__*Schizothecium* | -0.608* | 0.016 |
| g__*Pseudoxanthomonas* | -0.617* | 0.014 |  | g__*Tausonia* | 0.605* | 0.017 |
| g__*Pedobacter* | -0.604* | 0.017 |  | g__*Waitea* | -0.589* | 0.021 |
| g__UTBCD1 | 0.6* | 0.018 |  | g__*Sarocladium* | -0.577* | 0.024 |
| g__*Marmoricola* | 0.589* | 0.021 |  | g__*Filobasidium* | -0.576* | 0.025 |
| g__*Opitutus* | -0.58* | 0.023 |  | g__*Limonomyces* | -0.557* | 0.031 |
| g__*Stenotrophobacter* | -0.577* | 0.024 |  | g__*Zopfiella* | -0.557* | 0.031 |
| g__WX53 | 0.574* | 0.025 |  | g__*Acremonium* | -0.556* | 0.031 |
| g__*Rhizobacter* | -0.557* | 0.031 |  | g__*Fusarium* | 0.552* | 0.033 |
| g__*Blastopirellula* | 0.557* | 0.031 |  | g__*Coniochaeta* | 0.543* | 0.036 |
| g__*Acidovorax* | -0.555* | 0.032 |  | g__*Aspergillus* | 0.539* | 0.038 |
| g__*Kaistia* | -0.552* | 0.033 |  | g__*Naganishia* | 0.532* | 0.041 |
| g__*Neorhizobium* | -0.552* | 0.033 |  | g__*Symmetrospora* | -0.522* | 0.046 |
| g__*Duganella* | 0.548* | 0.034 |  | g__*Torula* | -0.519* | 0.048 |
| g__*Paraburkholderia* | 0.546* | 0.035 |  |  |  |  |
| g__*Acinetobacter* | -0.545* | 0.036 |  |  |  |  |
| g__*Dyadobacter* | -0.529* | 0.043 |  |  |  |  |
| g__*Caballeronia* | 0.526* | 0.044 |  |  |  |  |
| g__*Oligoflexus* | -0.524* | 0.045 |  |  |  |  |
| g__*Microterricola* | -0.521* | 0.046 |  |  |  |  |
| g__OLB17 | 0.517* | 0.049 |  |  |  |  |
| g__*Lacunisphaera* | -0.516* | 0.049 |  |  |  |  |
| g__*Pantoea* | -0.515* | 0.05 |  |  |  |  |
| g__*Asticcacaulis* | -0.515* | 0.05 |  |  |  |  |
